# Association between insufficient sleep and mental health in preschoolers: effect modification by low household income

**DOI:** 10.3389/fpsyt.2026.1898794

**Published:** 2026-09-03

**Authors:** Guiting Ren, Jia Wang, Xi Zhang, Shuangyu Li, Mingyue Duan

**Affiliations:** 1 School of Humanities and Social Sciences, Xi’an Jiaotong University, Xi’an, Shaanxi, China; 2 Faculty of Humanities, Shangluo University, Shangluo, Shaanxi, China; 3 School of Politics, Law & Public Administration, Yan’an University, Yan’an, Shaanxi, China; 4 Department of Clinical Laboratory, Xi’an Children’s Hospital, Affiliated Children’s Hospital of Xi’an Jiaotong University, National Regional Children’s Medical Center (Northwest), Xi’an, Shaanxi, China; 5 Department of Social Medicine, School of Public Health, Shanxi Medical University, Taiyuan, China

**Keywords:** effect modification, insufficient sleep, mental health, preschool children, socioeconomic factors, western China

## Abstract

**Background:**

Early childhood mental health problems are prevalent worldwide and can have long-term adverse outcomes. Insufficient sleep has been linked to poorer mental health, but evidence from disadvantaged populations remains limited. We examined this association between insufficient sleep and mental health outcomes in western Chinese preschoolers and whether it varies by demographic and socioeconomic factors.

**Methods:**

21,366 children aged 3–6 were recruited using stratified cluster sampling from 189 public kindergartens in a non-capital city of western China. Parent-reported daily sleep duration was categorized as <8, 8–<10, and 10–13 hours per day (reference). Mental health was assessed using the Strengths and Difficulties Questionnaire (SDQ), with outcomes as total difficulties (TDS >14) and prosocial behavior (PB <6). Multivariable regression models were used to estimate odds ratios (ORs) and coefficients (β), adjusting for demographic and socioeconomic covariates. Subgroup and interaction analyses were performed to identify effect modification.

**Results:**

Among 21,366 children, 18.6% had elevated total difficulties (TDS >14). Compared with children sleeping 10–13 h/d, those sleeping 8–<10 h/d had 45% higher odds of TDS >14 (OR = 1.45), and those sleeping <8 h/d had three-fold higher odds (OR = 3.07). Similar patterns were observed for continuous SDQ scores and prosocial behavior. The association of insufficient sleep with total difficulties decreased gradually across low-income (OR = 1.64), middle-income (60,000–99,999 CNY, OR = 1.34) and high-income households (OR = 1.22; *P* for interaction = 0.0137), and the association with low prosocial behavior strengthened with age (OR = 1.16 at <4 years; 1.29 at age 4; 1.45 at ≥5 years; *P* for interaction = 0.0186).

**Conclusion:**

Insufficient sleep is associated with poorer mental health in preschoolers, with stronger association among children from low-income families. Promoting adequate sleep, particularly in disadvantaged households, could be an effective strategy to improve mental health equity in early childhood.

## Introduction

Mental health problems in early childhood—including emotional, behavioral, and social difficulties—represent a major global public health concern, with prevalence estimates of 10–20% (1–4). These difficulties impair children’s immediate well-being and raise long-term risks such as school dropout, substance misuse, and suicidal ideation in adolescence and adulthood (5–7). Beyond individual suffering, pediatric mental disorders impose heavy socioeconomic burdens that hinder social development (8). Recent work points to rising prevalence and widening mental health disparities driven by socioeconomic inequities, academic pressure and digital overexposure, highlighting the urgent need for coordinated policy interventions (9). In China, local epidemiological data reveal prominent mental health challenges among young children. One SDQ-based survey conducted in impoverished rural areas reported that up to 70% of preschoolers displayed mental health symptoms, with emotional (39%) and conduct problems (27%) being the most prevalent (10). A national clinical survey using DSM-5 diagnostic criteria further estimated that 17.5% of Chinese children met the threshold for clinical mental disorders (11). These figures highlight the critical need to identify modifiable early-life risk factors.

Sleep constitutes one such pivotal modifiable factor. Current guidelines recommend 10–13 hours of daily sleep for preschoolers, with shorter durations considered insufficient (12, 13). Sufficient sleep sustains cognitive processes, enhances executive function, and supports positive social interaction and psychosocial adjustment (14, 15). Nevertheless, insufficient sleep has become an underrecognized global public health issue. Population-based studies across multiple countries document steady declines in children’s sleep duration over recent decades (16). In China, sleep problems affect nearly 40% of the pediatric population (17, 18), with children in western China disproportionately affected, likely due to regional socioeconomic gaps and limited healthcare access (19).

Extensive literature provides consistent evidence linking suboptimal sleep to elevated mental health problems across childhood. Cross-sectional data of children aged 5–12 years demonstrate that shorter sleep duration was associated with increased internalizing symptoms (20). Longitudinal evidence from diverse populations reinforces this link: a Norwegian cohort of children aged 6–12 years using objective actigraphy found that shorter sleep duration predicted higher odds of psychiatric disorders (21); and a U.S. prospective study revealed that persistent insufficient sleep during the preschool and early school years predicted higher SDQ total difficulties scores in mid-childhood (22).

Further research has examined whether socioeconomic status (SES) modifies the link between sleep and mental health. A recent meta-analysis of 104 studies (N = 326,478) demonstrated that SES acts as a moderator rather than merely a confounding covariate in this association, with stronger effects observed in low-SES samples for sleep consistency, and in middle-SES samples for objective sleep quality (23). Complementing this meta-analytic evidence, a narrative synthesis of the literature concluded that the association between poor sleep and psychosocial maladjustment is substantially stronger among children from lower-SES families, whereas higher-resource households provide protective buffering that attenuates such correlations (24).

Two complementary theoretical frameworks provide interpretive foundations for this interplay: the social determinants of health framework and the cumulative risk model. Guided by the social determinants of health framework, socioeconomic status (SES) is understood as a stratified social position generated by macroeconomic and political structural forces, rather than merely a demographic adjustment variable. As a key upstream determinant, SES shapes a suite of intermediate household and community contexts, including material resources, caregiving arrangements and residential environments, which collectively regulate children’s sleep and socioemotional growth (25, 26). The cumulative risk model elaborates that socioeconomic disadvantage accumulates multiple interrelated adversities, and each additional risk factor such as poor sleep can compound negative developmental outcomes, while higher SES offers protective resources to offset such cumulative harm (27).

However, despite theoretical and empirical support for SES stratification of sleep-mental health associations, analytical gaps persist within the existing research. First, the consistency of the SES-moderation effect should not be assumed across all samples; some investigations yield null interaction results when stratifying solely by household income, with no detectable SES differentiation in sleep-internalizing symptom correlations (28). These discrepancies may arise from methodological and contextual differences across studies, including variations in child age ranges, SES measurement approaches, and local socioeconomic and cultural environments. Second, few studies test SES as an interaction moderator rather than merely adjusting for it as a covariate (23). For example, a population-based study in Mongolia examined SES and sleep as independent risk factors in separate regression models without formally testing the interaction between the two (29). Third, most existing evidence on socioeconomic status as a moderator of sleep–mental health association originates from high-income Western populations (30), while comparable large-scale data from low- and middle-income countries remain limited.

In China, a lower-middle-income country with substantial regional disparities, several domestic studies have explored sleep-mental health correlations across Chinese regions. For example, large surveys of Shanghai preschoolers have documented significant linkages between sleep problems and prosocial behavior problems (31), and a cross-sectional analysis of 18,000 rural adolescents in Gansu Province detected nonlinear sleep–mental health relationships (32). However, large population-based datasets focusing exclusively on western Chinese preschoolers living under distinct socioeconomic disadvantages are still absent. Evidence from this non-capital western Chinese city sample can offer transferable references for disadvantaged preschool populations in resource-limited regions worldwide, though full cross-context generalization should be avoided. Therefore, this study aimed to (1) investigate the association between insufficient sleep and mental health outcomes among western Chinese preschoolers, and (2) examine whether demographic and socioeconomic factors modify these associations to identify high-risk subgroups and inform targeted interventions.

## Methods

### Study design and participants

A stratified cluster random sampling method was employed to recruit preschool children aged 3–6 years from a non-capital city located in Shaanxi Province, western China. According to the *China Statistical Yearbook 2024* (33), Shaanxi’s per capita disposable income of residents was 33,905 CNY in 2024, equivalent to 82.1% of the national average (41,314 CNY). As a non-capital city in northern Shaanxi, the local economy relies heavily on the energy industry, which elevates its per capita GDP but does not correspondingly raises household disposable income levels.

All 13 administrative districts and counties within the city were incorporated into the sampling frame, with public preschools randomly selected proportional to the regional population density of preschool-aged children. Children from junior, middle, and senior kindergarten classes were invited to participate after obtaining approval from school principals. The study objectives were explained, and written informed consent was obtained prior to data collection. Data were collected between February 28 and March 5, 2025, with coordination by the local Education Bureau and administration through the preschools. Questionnaires were distributed by classroom teachers; parents were given 2–3 days to complete the questionnaires at home and return them to the teachers. The questionnaire was completed by the primary parent—defined as the biological parent or legal guardian primarily responsible for the child’s daily care—who reported on the child’s sleep, mental health, and family background. This definition excluded broader caregivers (e.g., grandparents or nannies) to ensure reliable reporting of parent-specific constructs.

Of the 25,017 dyads initially enrolled, 2,408 were excluded as the respondent was a non-parent caregiver, leaving 22,609 eligible dyads. Among these, a further 1,243 were excluded due to missing or implausible child or parent age data, resulting in a final analytical sample of 21,366 dyads (**Supplementary Figure 1**).

The study was approved by the Ethics Committee of the Affiliated Children’s Hospital of Xi’an Jiaotong University (No. 20250225-21) and followed the Declaration of Helsinki.

### Assessment of sleep duration

Parental reports were used to estimate children’s daily sleep duration with the question: “On average, how many hours did your child sleep per day (including daytime naps and nighttime sleep) over the past month?” Responses were grouped into <8, 8–<10, 10–13 hours per day (h/d). Consistent with current guidelines (12, 13), sleep duration was also dichotomized as insufficient (<10 h/d) versus sufficient (10–13 h/d) for subgroup analyses.

### Assessment of mental health

Children’s mental health was assessed using the Strengths and Difficulties Questionnaire (SDQ), a validated instrument widely used to assess children’s mental health (34, 35). The SDQ consists of 25 items rated on a 3-point scale (0 = Not true, 1 = Somewhat true, 2 = Certainly true). The total difficulties score (TDS) was calculated by summing four subscales: emotional symptoms (5 items), conduct problems (5 items), hyperactivity/inattention (5 items), and peer relationship problems (5 items), yielding a range of 0–40, with higher scores indicating greater difficulties. The internalizing problems domain comprises the emotional symptoms and peer problems subscales, while the externalizing problems domain comprises the conduct problems and hyperactivity/inattention subscales (36). The prosocial behavior (PB) subscale comprises the remaining 5 items and measures positive social behaviors such as sharing and helping, with higher scores reflecting better prosocial functioning (range 0–10). Based on validated Chinese normative cut-offs for children aged 3–17 years (37), a TDS >14 was defined as elevated risk for mental health problems, and a PB score <6 was defined as unusually low prosocial behavior. In the present sample, the TDS and PB both showed acceptable internal consistency (Cronbach’s α = 0.70 for TDS and 0.83 for PB).

### Covariates

At baseline, demographic and socioeconomic characteristics of the participating families were collected. Child age and parent age were recorded as continuous variables (in years). Binary variables comprised child gender (boy/girl), parent gender (male/female), single-child status (yes/no), parental employment (employed/unemployed), and marital status (married vs. unmarried; unmarried included cohabitating, divorced, separated, widowed, and never-married). Parental education was grouped into junior high school or below, high school or junior college, and bachelor’s degree or above. Housing situation was categorized as owned outright, owned with mortgage, renting, or cohabiting with relatives.

Annual household income was collected via eight predefined bands in the questionnaire. To guide income categorization, we first examined the pattern of sleep–TDS associations across the original eight income bands (**Supplementary Table 1**). The ORs were consistently elevated (~1.6) for all bands below 60,000 CNY, but declined markedly (~1.3) at and above 60,000 CNY, suggesting a natural threshold. With reference to China’s National Bureau of Statistics report (33), the official data classify residents into five income quintiles, with the middle quintile threshold at 32,195 CNY and the high quintile boundary at 95,055 CNY—to which our 60,000 CNY and 100,000 CNY cut-offs approximately correspond. Based on the empirical pattern and national income distribution data, we adopted a three-category classification: low-income (<60,000 CNY), middle-income (60,000–99,999 CNY), and high-income (≥100,000 CNY). The ≥100,000 CNY cut-off was selected to further distinguish middle- from high-income families while maintaining sufficient sample sizes for stable stratified analyses.

### Statistical analysis

Given the potential clustering of children within kindergartens, we first estimated the intraclass correlation coefficient (ICC) for each outcome using a null model with kindergarten as a random intercept. The ICC for total difficulties and prosocial behavior were effectively zero, indicating no meaningful clustering effect (**Supplementary Table 2**). Therefore, standard logistic regression models without random effects were deemed appropriate for the primary analyses. To assess multicollinearity, we computed variance inflation factors (VIF) for all independent variables included in the final regression models. All VIF values were below the threshold of 5 (range: 1.0–1.3), indicating no substantial multicollinearity (**Supplementary Table 3**).

Continuous variables were assessed for normality using the Anderson–Darling test and were reported as the means ± standard deviations (SD) (normal) or median (interquartile range, IQR) (non-normal), whereas categorical variables were presented as counts (percentages). For baseline comparisons, children were stratified by total difficulties score (TDS >14 vs. ≤14) and prosocial behavior score (<6 vs. ≥6). Between-group differences were tested using independent Student’s t-test for normally distributed continuous variables and the Mann–Whitney U test for non-normally distributed variables; categorical variables were compared using the chi-square test or Fisher’s exact test as appropriate.

Binary outcomes were analyzed using multivariable logistic regression, with results reported as odds ratios (ORs) and 95% confidence intervals (CIs). Continuous outcomes, including total difficulties score, prosocial behavior score, and the four SDQ subscales (emotional symptoms, conduct problems, hyperactivity/inattention, peer problems), were analyzed using multivariable linear regression, with results presented as regression coefficients (β) and 95% CIs. For the four SDQ subscales, we applied permutation-based correction for multiple comparisons (1,000 resamples) to obtain empirical P values (38). An empirical P < 0.05 was considered statistically significant. Three hierarchical models were fitted: Model I was unadjusted; Model II was adjusted for child age/parent gender and age; Model III was additionally adjusted for all other covariates, including single-child status, marital status, annual household income, parental education level, employment status, and housing situation.

To evaluate potential effect modification, we performed subgroup analyses stratified by each demographic and socioeconomic variable. Interaction was formally tested by adding a product term (sleep duration × stratification variable) to the fully adjusted logistic regression model, with significance determined by the likelihood ratio test.

Missing or implausible data were present for child and parent age (n = 1,243, 5.50% of the eligible sample). To address this and examine robustness, we employed multiple imputation using chained equations (MICE), generating five imputed datasets (39). The imputation model included all covariates used in the primary analyses. Rubin’s rules were applied to combine the estimates across the imputed datasets (40).

All analyses were performed using R software (Version 4.4.1, http://www.R-project.org, R Foundation) and EmpowerStats (http://www.empowerstats.com, X&Y Solutions, Inc., Boston, MA). A two-sided *P* value < 0.05 was considered statistically significant.

## Results

### Demographic characteristics

Of the 21,366 children (median age 4.90 [IQR 1.50] years; 51.77% boys), 3,978 (18.62%) were at risk for emotional and behavioral problems (TDS >14). Most children slept 8–<10 h/d (68.60%), while only 28.74% met the recommended 10–13 h/d. The at-risk group had a lower proportion of sufficient sleep than the normal group (21.74% vs. 30.34%, *P* < 0.001). Compared with the normal group, the at-risk group had significantly higher proportions of boys, lower family income, lower parental education, lower employment rate, and more residential instability (renting or cohabiting with relatives) (all *P* < 0.001). No significant differences were observed for child age or single-child status (both *P* > 0.05) (**Table 1**).

**Table 1:** Characteristics of study participants by total difficulties score.

Variables	N = 21,366	TDS ≤14 N = 17,388	TDS >14 N = 3,978	*P*-value
Child age (years)	4.90 (1.50)	4.90 (1.50)	4.90 (1.60)	0.222
Primary parent age (years)	34.50 (5.50)	34.50 (5.50)	34.20 (5.58)	<0.001
Child gender				<0.001
Boys	11,062 (51.77%)	8,897 (51.17%)	2,165 (54.42%)	
Girls	10,304 (48.23%)	8,491 (48.83%)	1,813 (45.58%)	
Primary parent gender				<0.001
Male	5,108 (23.91%)	3,961 (22.78%)	1,147 (28.83%)	
Female	16,258 (76.09%)	13,427 (77.22%)	2,831 (71.17%)	
Single child status				0.386
Yes	6,148 (28.77%)	4,981 (28.65%)	1,167 (29.34%)	
No	15,218 (71.23%)	12,407 (71.35%)	2,811 (70.66%)	
Marital status				<0.001
Married	20,720 (96.98%)	16,906 (97.23%)	3,814 (95.88%)	
Unmarried	646 (3.02%)	482 (2.77%)	164 (4.12%)	
Annual household income (thousands, CNY)				<0.001
< 60	13,024 (61.0%)	10,261 (59.0%)	2,763 (69.5%)	
60-99	4,214 (19.7%)	3,559 (20.5%)	655 (16.5%)	
≥ 100	4,128 (19.3%)	3,568 (20.5%)	560 (14.1%)	
Parental education level				<0.001
Junior high school or below	5,741 (26.87%)	4,337 (24.94%)	1,404 (35.29%)	
High school or junior college	9,476 (44.35%)	7800 (44.86%)	1,676 (42.13%)	
Bachelor’s degree or above	6,149 (28.78%)	5251 (30.20%)	898 (22.57%)	
Parental employment status				<0.001
Employed	15,642 (73.21%)	12,912 (74.26%)	2,730 (68.63%)	
Unemployed	5724 (26.79%)	4476 (25.74%)	1248 (31.37%)	
Housing situation				<0.001
Owned outright	11,577 (54.18%)	9,626 (55.36%)	1,951 (49.04%)	
Owned with mortgage	3,391 (15.87%)	2,781 (15.99%)	610 (15.33%)	
Renting	4,412 (20.65%)	3,451 (19.85%)	961 (24.16%)	
Cohabiting with relatives	1,986 (9.30%)	1,530 (8.80%)	456 (11.46%)	
Sleep duration (h/d)				<0.001
Sufficient sleep (10–13 h/d)	6,140 (28.74%)	5,275 (30.34%)	865 (21.74%)	
Insufficient sleep (h/d)	15,226 (71.26%)	12,113 (69.66%)	3,113 (78.26%)	
<8	569 (2.66%)	357 (2.05%)	212 (5.33%)	
8–<10	14,657 (68.60%)	11,756 (67.61%)	2,901 (72.93%)	

SDQ Total Difficulties Score (TDS) was categorized as >14 (at risk for elevated difficulties) versus ≤14 (typical development). Median (interquartile range, IQR) for continuous variables; *P* value was calculated by the Mann–Whitney U test. N (%) for categorical variables; *P* value was calculated by the chi-square test or Fisher’s exact test as appropriate. A two-tailed P < 0.05 was considered statistically significant.

TDS, total difficulties score; h/d, hours/day; CNY, Chinese Yuan (to convert to US dollar, multiply by 6.80).

Baseline characteristics stratified by prosocial behavior status are presented in **Supplementary Table 4**. Compared with the adequate prosocial behavior group, those with low prosocial behavior (PB <6) were more often boys, less likely to have sufficient sleep, and had lower family income, lower parental education, and rented housing (all *P* < 0.001).

### Associations between sleep duration and mental health outcomes

The associations between sleep duration and mental health outcomes are summarized in **Tables 2**–**4**. Multivariable logistic regression analyses (**Table 2**) showed that insufficient sleep was associated with significantly higher odds of both elevated total difficulties and low prosocial behavior in a graded manner. After full adjustment (Model III), children sleeping 8–<10 h/d had 45% higher odds of total difficulties at risk (OR = 1.45, 95% CI: 1.33–1.57, *P* < 0.0001), and those sleeping <8 h/d had a three-fold increased risk (OR = 3.08, 95% CI: 2.54–3.71, *P* < 0.0001). For low prosocial behavior, the corresponding odds were 29% higher for the 8–<10 h/d group (OR = 1.29, 95% CI: 1.21–1.37, *P* < 0.0001) and more than 2-fold higher for the <8 h/d group (OR = 2.37, 95% CI: 1.97–2.84, *P* < 0.0001). When analyzed as continuous outcomes (**Table 3**), insufficient sleep was associated with higher total difficulties scores (8–<10 h/d: β = 0.83, 95% CI: 0.68–0.97; <8 h/d: β = 3.25, 95% CI: 2.83–3.67; both *P* < 0.0001) and lower prosocial behavior scores (8–<10 h/d: β = –0.30, 95% CI: –0.36 to –0.23; <8 h/d: β = –1.21, 95% CI: –1.40 to –1.03; both *P* < 0.0001).

**Table 2:** Multivariable logistic regression analysis of the association between sleep duration and mental health outcomes.

Outcomes	Model I		Model II		Model III	
	OR (95%CI)	*P*-Value	OR (95%CI)	*P*-Value	OR (95%CI)	P-Value
TDS >14						
Sleep duration (h/d)						
10–13	Ref.		Ref.		Ref.	
8–<10	1.50 (1.39, 1.63)	<0.0001	1.52 (1.40, 1.65)	<0.0001	1.45 (1.33, 1.57)	<0.0001
<8	3.62 (3.01, 4.36)	<0.0001	3.65 (3.03, 4.40)	<0.0001	3.07 (2.54, 3.70)	<0.0001
PB <6						
Sleep duration (h/d)						
10–13	Ref.		Ref.		Ref.	
8–<10	1.25 (1.17, 1.32)	<0.0001	1.32 (1.24, 1.40)	<0.0001	1.29 (1.21, 1.37)	<0.0001
<8	2.30 (1.92, 2.75)	<0.0001	2.47 (2.06, 2.97)	<0.0001	2.37 (1.97, 2.84)	<0.0001

Model I: adjusted for none. Model II: adjusted for child/parental age and gender. Model III: additionally adjusted for single-child status, marital status, annual household income, parental education level, employment status and housing situation. TDS >14 indicates elevated risk for mental health problems; PB <6 indicates low prosocial behavior.

OR, odds ratio; CI, confidence interval; h/d, hours/day.

**Table 3:** Multivariable linear regression analysis of the association between sleep duration and continuous mental health outcomes.

Sleep duration (h/d)	Total difficulties score		Prosocial behavior score	
	β(95%CI)	*P*-Value	β(95%CI)	*P*-Value
10–13	Ref.		Ref.	
8–<10	0.83 (0.68, 0.97)	<0.0001	-0.30 (-0.36, -0.23)	<0.0001
<8	3.25 (2.83, 3.67)	<0.0001	-1.21 (-1.40, -1.03)	<0.0001

Adjusted for all covariates as in **Table 2** Model III.

β, regression coefficient; CI, confidence interval; h/d, hours/day.

**Table 4:** Multivariable linear regression analysis of the association between sleep duration and the four SDQ subscales (continuous scores).

Sleep duration (h/d)	Emotional symptoms		Conduct problems		Hyperactivity/inattention		Peer problems	
	β(95%CI)	*P*-Value, empirical *P*	β(95%CI)	*P*-Value, empirical *P*	β(95%CI)	*P*-Value, empirical *P*	β(95%CI)	*P*-Value, empirical *P*
10–13	Ref.		Ref.		Ref.		Ref.	
8–<10	0.18 (0.13, 0.24)	<0.0001, 0.001	0.14 (0.10, 0.18)	<0.0001, 0.001	0.26 (0.21, 0.30)	<0.0001, 0.001	0.24 (0.18, 0.30)	<0.0001, 0.001
<8	0.70 (0.54, 0.86)	<0.0001, 0.001	0.77 (0.66, 0.89)	<0.0001, 0.001	1.09 (0.95, 1.22)	<0.0001, 0.001	0.69 (0.52, 0.86)	<0.0001, 0.001

Adjusted for all covariates as in **Table 2** Model III. Empirical *P* values were derived from permutation tests (1,000 resamples) for the four SDQ subscales.

β, regression coefficient; CI, confidence interval; h/d, hours/day.

To assess the robustness of our findings and examine domain-specific associations, we analyzed the four SDQ subscales as continuous outcomes with permutation-based correction for multiple comparisons (**Table 4**). Insufficient sleep was significantly associated with higher scores on all four subscales in a graded manner. After correction, all associations remained statistically significant (empirical *P* = 0.001 for all). The largest effect was observed for hyperactivity/inattention (β = 1.09 for <8 h/d), followed by conduct problems (β = 0.77), emotional symptoms (β = 0.70), and peer problems (β = 0.69).

### Subgroup and interaction analyses

**Figure 1:**
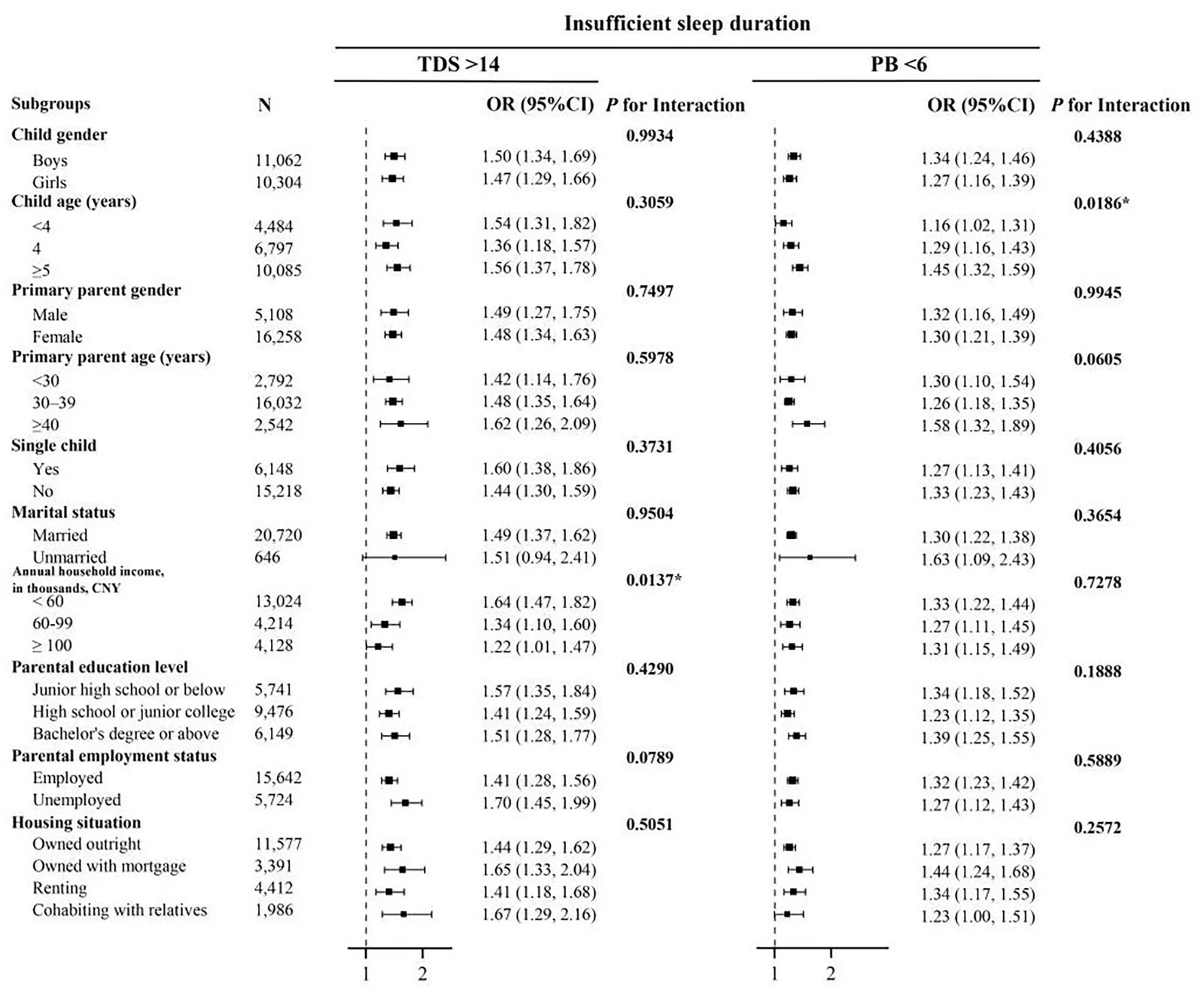
Subgroup and interaction analyses of the association between insufficient sleep and mental health outcomes by demographic and socioeconomic factors. Adjusted for all covariates as in **Table 2** Model III. In each case, the model was not adjusted for the stratification variable itself. Sleep duration was dichotomized as insufficient (<10 h/d) vs sufficient (10–13 h/d) for subgroup and interaction analyses. ORs and 95% CIs are shown for each subgroup; *P* for interaction were derived from likelihood ratio tests. TDS >14 indicates elevated risk for mental health problems; PB <6 indicates low prosocial behavior. OR, odds ratio; CI, confidence interval; CNY, Chinese Yuan (to convert to US dollar, multiply by 6.80).

Subgroup and interaction analyses were conducted to examine whether the association between insufficient sleep and mental health outcomes varied across demographic and socioeconomic factors (**Figure 1**). For total difficulties, significant effect modification was observed for annual household income (*P* for interaction =0.0137). The association was strongest in low-income households (<60,000 CNY: OR = 1.64, 95% CI: 1.47–1.82), attenuated in middle-income households (60,000–99,999 CNY: OR = 1.34, 95% CI: 1.10–1.60), and weakest in high-income households (≥100,000 CNY: OR = 1.22, 95% CI: 1.01–1.47), showing a clear graded pattern. For prosocial behavior, child age was a significant effect modifier (*P* for interaction = 0.0186). The association strengthened progressively with increasing age: OR = 1.16 (95% CI: 1.02–1.31) for children aged <4 years, 1.29 (95% CI: 1.16–1.43) for those aged 4 years, and 1.45 (95% CI: 1.32–1.59) for those aged ≥5 years. No significant interactions were detected for the remaining variables including child sex, parental age and gender, single-child status, marital status, parental education, employment status, and housing situation (all *P* for interaction > 0.05).

### Sensitivity analyses

To assess robustness of our findings, multiple imputation was performed for missing or implausible child and parent age data (n = 1,243). The pooled estimates from five imputed datasets were consistent with the complete-case analysis (**Supplementary Table 5**), indicating that missing data did not substantially bias the results. To examine whether the observed associations were driven by children with extremely short sleep duration, we repeated the logistic regression after excluding children sleeping <8 h/d (n = 569) (**Supplementary Table 6**). The estimates remained nearly identical to those from the full sample, confirming that the findings were not driven by the small subgroup of extremely short sleepers.

## Discussion

Using a sample of 21,366 children aged 3–6 years, we found a consistent, graded association: insufficient sleep was linked to higher odds of emotional and behavioral problems and lower prosocial behavior. The association between insufficient sleep and total difficulties was more pronounced in low-income families, and the association with low prosocial behavior strengthened with child age. A striking finding is the very low prevalence of sufficient sleep in our sample—only 28.7% of children slept the recommended 10–13 hours per day. This rate is markedly lower than that reported in high-income countries, where over 80% of children meet the guidelines (41), but aligns with previous domestic reports (19). To understand this disparity, it is important to consider the socioeconomic context of our sample. Compared with developed eastern cities such as Shanghai, our participants lived in families with much lower income (80.7% vs. 21.0% with annual income <100,000 CNY), a lower proportion of single-child families (28.8% vs. 74.2%), and lower parental education attainment (e.g., parental university education: 28.7% vs. 56.9%) (42). These socioeconomic disadvantages likely contribute to the high prevalence of insufficient sleep in this region and may amplify its adverse mental health effects.

The graded association between insufficient sleep and poorer mental health in our study aligns with previous findings. Specifically, a meta-analysis of 37 studies demonstrated that sleep is directly associated with both internalizing and externalizing problems (43), and longitudinal evidence from rural China has shown that shorter nighttime sleep duration in preschoolers prospectively predicts higher SDQ scores (44). For prosocial behavior, a large-scale study of 23,524 Shanghai preschoolers aligns with our findings, showing that shorter nighttime sleep duration was associated with higher odds of prosocial behavior problems (31). Domain-specific analyses revealed that the strongest effect of insufficient sleep was on hyperactivity/inattention. This pattern aligns with existing evidence linking short sleep to ADHD-related difficulties as measured by clinically validated ADHD instruments: shorter sleep duration was associated with higher odds of ADHD symptoms (45), and treating sleep dysregulation improves core ADHD symptoms in children (46). Thus, insufficient sleep is broadly associated with child mental health, potentially with particular salience for attention-related difficulties.

Mechanistically, insufficient sleep may disrupt neurobehavioral function through impaired prefrontal cortex connectivity and dysregulated amygdala activity, exacerbating emotional and behavioral dysregulation (47, 48). Recent evidence suggests that insufficient sleep during early childhood is associated with alterations in cortical thickness, synaptic pruning, and executive-function maturation (49). Longitudinal data indicate that sleep disturbances serve as a mediator between neural connectivity anomalies and deteriorating youth mental health, with a general sleep disturbance factor predicting future internalizing and externalizing symptoms (50). Conversely, adequate sleep enhances functional connectivity in emotional regulation networks (51), promoting emotional stability, cognitive development, and overall mental well-being (15, 52). These neurobiological pathways provide a plausible explanation for the consistent associations observed across diverse sociodemographic strata.

Beyond the main association, subgroup and interaction analyses identified a clear income-graded pattern, with the association strongest in low-income households (OR = 1.64), intermediate in middle-income households (OR = 1.34), and weakest in high-income households (OR = 1.22; P for interaction = 0.0137). This pattern reflects that socioeconomic disadvantage increases children’s vulnerability to additional stressors, such as insufficient sleep. Longitudinal studies have confirmed that socioeconomic risks predict persistent sleep problem trajectories from infancy to school age (53). A cross-sectional study has also shown that cumulative risk exposures moderate the sleep-mental health association, such that children with the poorest sleep health behaviors and highest cumulative risks had the greatest internalizing problems (28).

Drawing on the social determinants of health framework (25), household income functions as a key structural determinant shaping multiple proximal mechanism—including family stress, home environment, bedtime routines, healthcare access, and caregiving capacity—that collectively shape children’s sleep and mental well-being. Consistent with the cumulative risk model (27), low-income families commonly experience multiple co-occurring stressors that jointly strain children’s physiological and psychological resilience (54–57). Within this framework, insufficient sleep acts as an additional risk burden, showing disproportionately stronger associations with adverse outcomes. Furthermore, the protective resources that could compensate for the risks linked to sleep loss (e.g., consistent bedtime routines, quiet sleep environments, nutritious meals, and engaged parenting) are often less available in disadvantaged households (58). In contrast, children in higher-income families benefit from a wider array of developmental resources that may partially mitigate the risks linked to insufficient sleep. Although they face distinct stressors, such as academic pressure and reduced parental involvement (59, 60), these pressures do not eliminate the overall protective buffer provided by other resources.

Complementing the social-ecological pathways above, neurobiological evidence provides supplementary explanatory support. A recent study found that income insufficiency predicts delayed brain maturation in infants (61), indicating that the developing nervous systems of children in low-income families may be inherently less resilient to the extra burden from insufficient sleep. Taken together, these multi-layered interpretations carry critical implications for early childhood health equity, and targeted sleep interventions in low-income communities could help reduce early-childhood disparities.

Cross-cultural evidence on SES as a moderator of the sleep–mental health association remains limited. In the available U.S.-based meta-analysis, subjective sleep duration showed a weaker association with mental health in low-SES samples compared to mixed-SES samples, suggesting that other adversities may attenuate its predictive power (23). In contrast, our findings from western China show a stronger association in low-income families. This divergence may reflect contextual differences in how socioeconomic disadvantage relates to sleep–mental health association. In the U.S., pervasive poverty-related stressors may overwhelm the sleep signal; in our sample, household income may more directly shape the resources that support both sleep and child mental health. This discrepancy underscores the need for large-scale evidence from understudied low-resource settings.

We also found that child age significantly modified the association between insufficient sleep and low prosocial behavior (*P* for interaction = 0.0186). The association became progressively stronger with increasing age (<4 years to ≥5 years). This age-dependent pattern can be explained by the developmental trajectory of prosocial behavior. During the preschool years, prosocial actions—such as sharing, helping, and comforting—shift from simple, sympathy-driven responses to more complex behaviors guided by social norms, cognitive maturation, and peer interactions (62–64).

A recent large-scale study reported that cognitive empathy—a capacity that gradually matures during early childhood—partially mediates the association between sleep and prosocial behaviors (31). It is plausible that older preschoolers, whose prosocial behaviors rely more on cognitive empathy, are more susceptible to sleep loss than younger children, whose prosocial actions are primarily driven by more simple responses than by complex cognitive processing. This finding does not imply that sleep is unimportant for younger children. Rather, it suggests that the consequences of sleep insufficiency for social development become increasingly evident as social expectations and cognitive demands increase during the preschool period.

This study provides several novel contributions to the literature. First, it is among the largest population-based investigations of sleep and mental health in Chinese preschoolers, drawing on a sample from a non-capital western city—a region largely underrepresented in prior research. Second, we formally tested household income as an effect modifier and found that low socioeconomic status strengthens the link between insufficient sleep and child mental health difficulties. These findings position insufficient sleep as a critical health equity concern shaped by household socioeconomic conditions, and are likely transferable to other low-resource regions with similar socioeconomic gradients. However, the extent of this transferability depends on local contextual factors—including economic conditions, family structure, and healthcare access—and should be assessed in future comparative research.

### Limitations

Several limitations should be acknowledged. First, the cross-sectional design precludes causal inference, as temporal ordering between insufficient sleep and child mental health outcomes cannot be established. Moreover, the cross-sectional nature of the data also leaves open the possibility of bidirectional relationships, and residual confounding from unmeasured household-level factors may have influenced the observed associations. Longitudinal data are needed to confirm causality and identify critical exposure windows (e.g., early vs. late preschool years). Second, both sleep duration and mental health outcomes were parent-reported, which may introduce same-source bias. Because the same respondent provided both exposure and outcome information, observed associations may be inflated by common rater effects. Reliance on a single informant also limits our ability to capture context-specific variations in child behavior (e.g., home vs. kindergarten). Future studies would benefit from multi-informant approaches (e.g., teacher-rated SDQ) and objective sleep monitoring (e.g., actigraphy) to mitigate this limitation. Third, several important confounders were not measured, including parental sleep behaviors, marital conflict, parenting style, as well as child and parental dietary patterns. These factors may relate to both child sleep and mental health, potentially leading to residual confounding. Fourth, we assessed sleep only as average total daily duration, without capturing sleep quality, bedtime regularity, bedtime screen exposure, or specific sleep disturbances. These unmeasured sleep characteristics are established correlates of early childhood mental health and could confound or modify the observed associations. Notably, we did not separately record nighttime sleep and in-kindergarten midday napping. Parents may lack precise information about school-based nap duration, potentially introducing measurement error in sleep assessment. The lack of differentiated sleep indicators limits the comprehensiveness of our sleep assessment and warrants improvement in future investigations. Finally, participants were recruited exclusively from a non-capital city in western China, which may limit generalizability to populations with different socioeconomic or cultural backgrounds. However, the large sample and stratified cluster sampling design enhance representativeness for similar underdeveloped regions in western China.

## Conclusions

This cross-sectional study provides consistent evidence that insufficient sleep is associated with higher odds of total difficulties and lower prosocial behavior in preschool children, showing a clear graded pattern. Notably, the association between insufficient sleep and total difficulties was the strongest in low-income households. These findings highlight that sleep interventions should not rely on one-size-fits-all universal schemes, but should be tailored to the socioeconomic conditions of families. Our results have implications for interventions at multiple levels—family, school, and primary health care—with priority given to low-income households. Practical strategies could include parental education on bedtime routines, reducing evening screen exposure, fostering kindergarten-family collaboration, and sleep screening in child health services, thereby helping to reduce disparities in early childhood mental health. Future longitudinal studies are warranted to verify causal pathways and evaluate the effectiveness of income-stratified sleep intervention programs for preschool children in western China.

## Data Availability

The raw data supporting the conclusions of this article will be made available by the authors, without undue reservation.

## References

[1] von Klitzing K Döhnert M Kroll M Grube M . Mental disorders in early childhood. Dtsch Arztebl Int. (2015) 112:375–86. doi: 10.3238/arztebl.2015.0375 26149380 PMC4496484

[2] Black MM Jukes MCH Willoughby MT . Behavioural and emotional problems in preschool children. Lancet Psychiatry. (2017) 4:89–90. doi: 10.1016/s2215-0366(17)30005-6 28137382

[3] Charach A Mohammadzadeh F Belanger SA Easson A Lipman EL McLennan JD . Identification of preschool children with mental health problems in primary care: Systematic review and meta-analysis. J Can Acad Child Adolesc Psychiatry. (2020) 29:76–105. 32405310 PMC7213917

[4] Kieling C Baker-Henningham H Belfer M Conti G Ertem I Omigbodun O . Child and adolescent mental health worldwide: Evidence for action. Lancet. (2011) 378:1515–25. doi: 10.1016/s0140-6736(11)60827-1 22008427

[5] Kessler RC Angermeyer M Anthony JC De Graaf R Demyttenaere K Gasquet I . Lifetime prevalence and age-of-onset distributions of mental disorders in the World Health Organization's World Mental Health Survey Initiative. World Psychiatry. (2007) 6:168–76. PMC2174588 18188442

[6] Dyer ML Easey KE Heron J Hickman M Munafò MR . Associations of child and adolescent anxiety with later alcohol use and disorders: A systematic review and meta-analysis of prospective cohort studies. Addiction. (2019) 114:968–82. doi: 10.1111/add.14575 30891835 PMC6563455

[7] World Health Organization . Comprehensive mental health action plan 2013-2030. (2021).

[8] Christensen MK Lim CCW Saha S Plana-Ripoll O Cannon D Presley F . The cost of mental disorders: A systematic review. Epidemiol Psychiatr Sci. (2020) 29:e161. doi: 10.1017/s204579602000075x 32807256 PMC7443800

[9] Cosma A Black M Vuckovic S Pavic I Fonseca H Lazzerini M . The changing epidemiology of child and adolescent mental health requires an immediate policy response. Public Health Pract (Oxf). (2025) 10:100655. doi: 10.1016/j.puhip.2025.100655 41113959 PMC12528862

[10] Li S Chen K Liu C Bi J He Z Luo R . Dietary diversity and mental health in preschoolers in rural China. Public Health Nutr. (2021) 24:1869–76. doi: 10.1017/s1368980020003237 33308358 PMC10195499

[11] Li F Cui Y Li Y Guo L Ke X Liu J . Prevalence of mental disorders in school children and adolescents in China: Diagnostic data from detailed clinical assessments of 17,524 individuals. J Child Psychol Psychiatry. (2022) 63:34–46. doi: 10.1111/jcpp.13445 34019305

[12] Hirshkowitz M Whiton K Albert SM Alessi C Bruni O DonCarlos L . National Sleep Foundation's sleep time duration recommendations: Methodology and results summary. Sleep Health. (2015) 1:40–3. doi: 10.1016/j.sleh.2014.12.010 29073412

[13] Paruthi S Brooks LJ D'Ambrosio C Hall WA Kotagal S Lloyd RM . Recommended amount of sleep for pediatric populations: A consensus statement of the American Academy of Sleep Medicine. J Clin Sleep Med. (2016) 12:785–6. doi: 10.5664/jcsm.5866 27250809 PMC4877308

[14] Short MA Carskadon MA . Sleep and mental health in children and adolescents. In: Grandner MA , editor. Sleep and Health. San Diego, CA: Academic Press (2019). p. 435–45.

[15] Baranwal N Yu PK Siegel NS . Sleep physiology, pathophysiology, and sleep hygiene. Prog Cardiovasc Dis. (2023) 77:59–69. doi: 10.1016/j.pcad.2023.02.005 36841492

[16] Matricciani L Olds T Petkov J . In search of lost sleep: Secular trends in the sleep time of school-aged children and adolescents. Sleep Med Rev. (2012) 16:203–11. doi: 10.1016/j.smrv.2011.03.005 21612957

[17] Cao H Yan S Gu C Wang S Ni L Tao H . Prevalence of attention-deficit/hyperactivity disorder symptoms and their associations with sleep schedules and sleep-related problems among preschoolers in Mainland China. BMC Pediatr. (2018) 18:70. doi: 10.1186/s12887-018-1022-1 29458356 PMC5817725

[18] Deng Y Zhang Z Gui Y Li W Rong T Jiang Y . Sleep disturbances and emotional and behavioral difficulties among preschool-aged children. JAMA Netw Open. (2023) 6:e2347623. doi: 10.1001/jamanetworkopen.2023.47623 38095895 PMC10722331

[19] Chen X Ke ZL Chen Y Lin X . The prevalence of sleep problems among children in Mainland China: A meta-analysis and systemic-analysis. Sleep Med. (2021) 83:248–55. doi: 10.1016/j.sleep.2021.04.014 34049044

[20] Wu LC Hattangadi N Keown-Stoneman CDG Maguire JL Birken CS Stremler R . Sleep duration and internalizing symptoms in children. J Can Acad Child Adolesc Psychiatry. (2022) 31:115–23. doi: 10.1093/pch/pxaa068.072 PMC9275369 35919906

[21] Ranum BM Wichstrøm L Pallesen S Falch-Madsen J Halse M Steinsbekk S . Association between objectively measured sleep duration and symptoms of psychiatric disorders in middle childhood. JAMA Netw Open. (2019) 2:e1918281. doi: 10.1001/jamanetworkopen.2019.18281 31880797 PMC6991225

[22] Taveras EM Rifas-Shiman SL Bub KL Gillman MW Oken E . Prospective study of insufficient sleep and neurobehavioral functioning among school-age children. Acad Pediatr. (2017) 17:625–32. doi: 10.1016/j.acap.2017.02.001 28189692 PMC5545152

[23] Thompson MJ Ehrhardt AD Saini EK Brigham EF Altinoz ZS Yip T . Unpacking sleep and mental health disparities across childhood and adolescence: A meta-analytic and systematic review. Sleep Med Rev. (2026) 85:102206. doi: 10.1016/j.smrv.2025.102206 41313963 PMC12853373

[24] El-Sheikh M Gillis BT Saini EK Erath SA Buckhalt JA . Sleep and disparities in child and adolescent development. Child Dev Perspect. (2022) 16:200–7. doi: 10.1111/cdep.12465 36337834 PMC9629655

[25] Solar O Irwin A . A conceptual framework for action on the social determinants of health (2010). Available online at: https://nccdh.ca/resources/entry/a-conceptual-framework/ (Accessed August 3, 2026).

[26] CSDH . Closing the Gap in a Generation: Health Equity Through Action on the Social Determinants of Health (2008). World Health Organization. Available online at: https://www.who.int/publications/i/item/WHO-IER-CSDH-08.1 (Accessed August 3, 2026).

[27] Evans GW Li D Whipple SS . Cumulative risk and child development. Psychol Bull. (2013) 139:1342–96. doi: 10.1037/a0031808 23566018

[28] Williamson AA Davenport M Cicalese O Mindell JA . Sleep problems, cumulative risks, and psychological functioning in early childhood. J Pediatr Psychol. (2021) 46:878–90. doi: 10.1093/jpepsy/jsab022 33738501 PMC8357222

[29] Aoki A Togoobaatar G Tseveenjav A Nyam N Zuunnast K Lkhagvasuren G . Socioeconomic and lifestyle factors associated with mental health problems among Mongolian elementary school children. Soc Psychiatry Psychiatr Epidemiol. (2022) 57:791–803. doi: 10.1007/s00127-021-02178-7 34595562 PMC8483169

[30] El-Sheikh M Shimizu M Philbrook LE Erath SA Buckhalt JA . Sleep and development in adolescence in the context of socioeconomic disadvantage. J Adolesc. (2020) 83:1–11. doi: 10.1016/j.adolescence.2020.06.006 32619770 PMC7484096

[31] Rong T Sun X Deng Y Liu J Lin Q Zhu Q . Sleep and prosocial behaviors among young preschoolers: The mediating role of cognitive empathy. Sleep Med. (2025) 131:106512. doi: 10.1016/j.sleep.2025.106512 40245792

[32] Liu W Guan H Chen X Zhang L . Insights into adolescent sleep and mental health in rural area of northwestern China. Sci Rep. (2024) 14:31082. doi: 10.1038/s41598-024-82220-1 39730730 PMC11680696

[33] China NBoSo . China statistical yearbook 2024 (2024). Available online at: https://www.stats.gov.cn/sj/ndsj/2024/indexch.htm (Accessed August 3, 2026).

[34] Goodman R . The Strengths and Difficulties Questionnaire: A research note. J Child Psychol Psychiatry. (1997) 38:581–6. doi: 10.1111/j.1469-7610.1997.tb01545.x 9255702

[35] Goodman A Goodman R . Strengths and Difficulties Questionnaire as a dimensional measure of child mental health. J Am Acad Child Adolesc Psychiatry. (2009) 48:400–3. doi: 10.1097/CHI.0b013e3181985068 19242383

[36] Goodman A Lamping DL Ploubidis GB . When to use broader internalising and externalising subscales instead of the hypothesised five subscales on the Strengths and Difficulties Questionnaire (SDQ): Data from British parents, teachers and children. J Abnorm Child Psychol. (2010) 38:1179–91. doi: 10.1007/s10802-010-9434-x 20623175

[37] Du Y Kou J Coghill D . The validity, reliability and normative scores of the parent, teacher and self report versions of the Strengths and Difficulties Questionnaire in China. Child Adolesc Psychiatry Ment Health. (2008) 2:8. doi: 10.1186/1753-2000-2-8 18445259 PMC2409296

[38] North BV Curtis D Sham PC . A note on the calculation of empirical *P* values from Monte Carlo procedures. Am J Hum Genet. (2002) 71:439–41. doi: 10.1086/341527 12111669 PMC379178

[39] van Buuren S Groothuis-Oudshoorn K . Mice: Multivariate imputation by chained equations in R. J Stat Software. (2011) 45:1–67. doi: 10.18637/jss.v045.i03

[40] Rubin DB . Multiple Imputation for Nonresponse in Surveys. Hoboken, NJ: John Wiley & Sons (1987).

[41] Cliff DP McNeill J Vella SA Howard SJ Santos R Batterham M . Adherence to 24-hour movement guidelines for the early years and associations with social-cognitive development among Australian preschool children. BMC Public Health. (2017) 17:857. doi: 10.1186/s12889-017-4858-7 29219104 PMC5773906

[42] Wang H Zhao J Yu Z Pan H Wu S Zhu Q . Types of on-screen content and mental health in kindergarten children. JAMA Pediatr. (2024) 178:125–32. doi: 10.1001/jamapediatrics.2023.5220 38048076 PMC10696513

[43] Vazsonyi AT Tehrani HD Yamini S . The links between sleep, self-control, and internalizing/externalizing problems: A meta-analysis. J Criminal Justice. (2025) 98:102416. doi: 10.1016/j.jcrimjus.2025.102416 42574925

[44] Liu H Ma S Feng L Gao J Wu B Xia W . Longitudinal association of nighttime sleep duration with emotional and behavioral problems among rural preschool children. Eur Child Adolesc Psychiatry. (2024) 33:267–77. doi: 10.1007/s00787-023-02153-4 36781466 PMC9925221

[45] Jiang H Yu B Liu Y Gao H Song R Tan S . Associations of lamplight exposure during sleep and sleep duration with attention-deficit/hyperactivity disorder among preschool children in China. Front Psychiatry. (2024) 15:1489229. doi: 10.3389/fpsyt.2024.1489229 39845361 PMC11751368

[46] Lunsford-Avery JR Wu JQ French A Davis NO . Topical review: Sleep regulation as a novel target for treating preschool-aged children with attention-deficit/hyperactivity disorder symptoms. J Pediatr Psychol. (2025) 50:266–71. doi: 10.1093/jpepsy/jsae107 39774675 PMC11981054

[47] Ben Simon E Vallat R Barnes CM Walker MP . Sleep loss and the socio-emotional brain. Trends Cognit Sci. (2020) 24:435–50. doi: 10.1016/j.tics.2020.02.003 32299657

[48] Khan MA Al-Jahdali H . The consequences of sleep deprivation on cognitive performance. Neurosci (Riyadh). (2023) 28:91–9. doi: 10.17712/nsj.2023.2.20220108 37045455 PMC10155483

[49] Anvarov F . The physiological importance of sleep and its impact on brain development in preschool-aged children. Int J Pedagogics. (2025) 5:35–7. doi: 10.37547/ijp/volume05issue06-10 42609749

[50] Wang Y Tahmasian M Genon S Samea F He Z Liu X . Multimodal neuroimaging signature of sleep problems predicts preadolescent mental health trajectories. Medrxiv. (2025). doi: 10.1101/2025.09.22.25336312 41040704 PMC12485969

[51] Killgore WD . Self-reported sleep correlates with prefrontal-amygdala functional connectivity and emotional functioning. Sleep. (2013) 36:1597–608. doi: 10.5665/sleep.3106 24179291 PMC3792375

[52] Scott AJ Webb TL Martyn-St James M Rowse G Weich S . Improving sleep quality leads to better mental health: A meta-analysis of randomised controlled trials. Sleep Med Rev. (2021) 60:101556. doi: 10.1016/j.smrv.2021.101556 34607184 PMC8651630

[53] Williamson AA Mindell JA Hiscock H Quach J . Sleep problem trajectories and cumulative socio-ecological risks: Birth to school-age. J Pediatr. (2019) 215:229–237.e4. doi: 10.1016/j.jpeds.2019.07.055 31564429 PMC6878157

[54] Chaudry A Wimer C . Poverty is not just an indicator: The relationship between income, poverty, and child well-being. Acad Pediatr. (2016) 16:S23–9. doi: 10.1016/j.acap.2015.12.010 27044698

[55] Yamaoka Y Isumi A Doi S Ochi M Fujiwara T . Differential effects of multiple dimensions of poverty on child behavioral problems: Results from the a-Child study. Int J Environ Res Public Health. (2021) 18:11821. doi: 10.3390/ijerph182211821 34831578 PMC8624981

[56] Khin YP Yamaoka Y Abe A Fujiwara T . Association of child-specific and household material deprivation with depression among elementary and middle school students in Japan. Soc Psychiatry Psychiatr Epidemiol. (2024) 59:329–39. doi: 10.1007/s00127-023-02502-3 37270468 PMC10239275

[57] Near CE . Mediators of the relation of family income with adolescent behavior problems and cognitive achievement: Material hardship, parent distress and parent support. Infant Child Dev. (2022) 31:e2316. doi: 10.1002/icd.2316 36590924 PMC9797181

[58] Bathory E Tomopoulos S . Sleep regulation, physiology and development, sleep duration and patterns, and sleep hygiene in infants, toddlers, and preschool-age children. Curr Probl Pediatr Adolesc Health Care. (2017) 47:29–42. doi: 10.1016/j.cppeds.2016.12.001 28117135

[59] Cong X Hosler AS Tracy M Appleton AA . The relationship between parental involvement in childhood and depression in early adulthood. J Affect Disord. (2020) 273:173–82. doi: 10.1016/j.jad.2020.03.108 32421599

[60] Steare T Gutiérrez Muñoz C Sullivan A Lewis G . The association between academic pressure and adolescent mental health problems: A systematic review. J Affect Disord. (2023) 339:302–17. doi: 10.1016/j.jad.2023.07.028 37437728

[61] Chung H Wilkinson CL Liu A Job Said A Francis B Cañaveral G . Income insufficiency impacts early brain development in infants facing increased psychosocial adversity: A network-based approach. Proc Natl Acad Sci USA. (2026) 123:e2513598123. doi: 10.1073/pnas.2513598123 41490482 PMC12799155

[62] Te Brinke LW van de Groep S van der Cruijsen R Crone EA . Variability and change in adolescents' prosocial behavior across multiple time scales. J Res Adolesc. (2023) 33:575–90. doi: 10.1111/jora.12827 36639955

[63] Grueneisen S Warneken F . The development of prosocial behavior-from sympathy to strategy. Curr Opin Psychol. (2022) 43:323–8. doi: 10.1016/j.copsyc.2021.08.005 34530222

[64] Eisenberg N Spinrad TL . Prosocial development. In: Damon W Lerner RM , editors. Handbook of Child Psychology: Social, Emotional, and Personality Development, 6th ed. Hoboken, NJ: John Wiley & Sons, Inc (2006). p. 646–718.

