# Supplementary material for: Association between insufficient sleep and mental health in preschoolers: effect modification by low household income
Source: Front Psychiatry. 2026 Sep 3;17:1898794. doi: 10.3389/fpsyt.2026.1898794 (PMC13582533; doi:10.3389/fpsyt.2026.1898794)
Supplement: Supplementary file 1 [file DataSheet1.pdf]

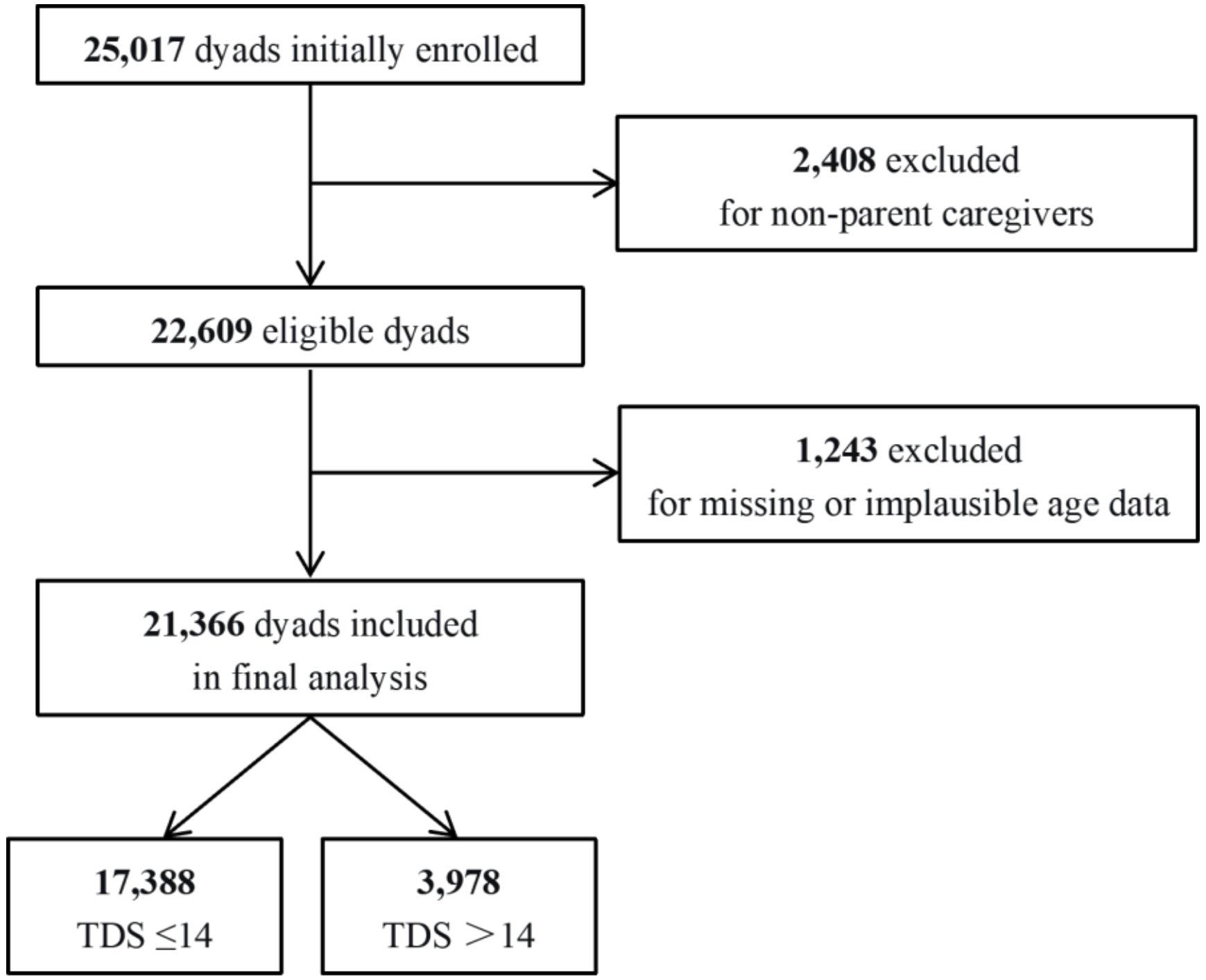

Supplementary Figure S1 Flowchart for sample selection.

Supplementary Table S1 Association between insufficient sleep and total difficulties stratified by the original eight household annual income bands.

Annual household income (CNY)	N	OR (95% CI)	*P*-value
<10,000	4,586	1.65 (1.38–1.97)	<0.0001
10,000–29,999	4,033	1.62 (1.35–1.94)	<0.0001
30,000–59,999	4,405	1.64 (1.36–1.99)	<0.0001
60,000–99,999	4,214	1.34 (1.10–1.60)	0.0020
100,000–149,999	2,577	1.25 (1.02–1.56)	0.0406
150,000–199,999	943	1.26 (0.79–1.88)	0.1049
200,000–299,999	372	1.04 (0.53–1.94)	0.8089
≥300,000	236	0.65 (0.28–1.47)	0.4057

Adjusted for all covariates as in Table 2 Model III, except for annual household income itself. The global interaction across the eight income bands was not statistically significant ($P = 0.2716$), likely reflecting limited statistical power in higher income subgroups due to small sample sizes. Sleep duration was dichotomized as insufficient (<10 h/d) vs sufficient (10–13 h/d).

Abbreviations: OR, odds ratio; CI, confidence interval; CNY, Chinese Yuan (to convert to US dollar, multiply by 6.80).

Supplementary Table S2 Variance components and intraclass correlation coefficients (ICC) from null models with kindergarten as random intercept.

Outcome	Between-kindergarten variance	Within-kindergarten variance	ICC	Model status
Total difficulties	4.64×10^{-14}	0.1515	< 0.001	Singular fit
Prosocial behavior	0.00	0.2500	0.000	Singular fit

ICC = between-kindergarten variance / (between-kindergarten variance + within-kindergarten variance). A singular fit indicates that the estimated between-kindergarten variance is effectively zero, suggesting no meaningful clustering.

Supplementary Table S3 Variance inflation factor (VIF) analysis for multicollinearity assessment.

Variable	VIF
Sleep duration	1
Child gender	1
Child age	1
Primary parent gender	1.1
Primary parent age	1.1
Single-child status	1.1
Marital status	1
Annual household income	1.2
Parental education level	1.3
Parental employment status	1.3
Housing situation	1.1

VIF = variance inflation factor, calculated as $1/(1-R^2)$. Stepwise screening was applied with a threshold of 5. All variables exhibited VIF < 5, indicating no substantial multicollinearity.

Table S4 Characteristics of study participants by prosocial behavior score.

Variables	PB ≥6<br>N = 10,808	PB <6<br>N = 10,558	*P*-value
**Child gender**			<0.001
Boys	5350 (49.50%)	5712 (54.10%)	
Girls	5458 (50.50%)	4846 (45.90%)	
**Child age (years)**	5.00 (1.40)	4.80 (1.50)	<0.001
**Primary parent gender**			<0.001
Male	2316 (21.43%)	2792 (26.44%)	
Female	8492 (78.57%)	7766 (73.56%)	
**Primary parent age (years)**	34.50 (5.60)	34.40 (5.40)	<0.001
**Single child status**			0.183
Yes	3154 (29.18%)	2994 (28.36%)	
No	7654 (70.82%)	7564 (71.64%)	
**Marital status**			0.053
Married	10457 (96.75%)	10263 (97.21%)	
Unmarried	351 (3.25%)	295 (2.79%)	
**Annual household income (thousands, CNY)**			<0.001
< 60	6411 (59.3%)	6613 (62.6%)	
60-99	2130 (19.7%)	2084 (19.7%)	
≥ 100	2267 (21.0%)	1861 (17.6%)	
**Parental education level**			<0.001
Junior high school or below	2740 (25.35%)	3001 (28.42%)	
High school or junior college	4830 (44.69%)	4646 (44.00%)	
University bachelor's degree or above	3238 (29.96%)	2911 (27.57%)	

**Parental employment status**			0.142
Employed	7960 (73.65%)	7682 (72.76%)	
Unemployed	2848 (26.35%)	2876 (27.24%)	
**Housing situation**			0.023
Owned outright	5918 (54.76%)	5659 (53.60%)	
Owned with mortgage	1729 (16.00%)	1662 (15.74%)	
Renting	2141 (19.81%)	2271 (21.51%)	
Cohabiting with relatives	1020 (9.44%)	966 (9.15%)	
**Sleep duration (h/d)**			<0.001
Sufficient sleep (10–13 h/d)	3370 (31.18%)	2770 (26.24%)	
Insufficient sleep (h/d)	7438 (68.82%)	7788 (73.76%)	
<8	197 (1.82%)	372 (3.52%)	
8–<10	7241 (67.00%)	7416 (70.24%)	

SDQ Prosocial Behavior (PB) score was categorized as <6 (at risk for low prosocial behavior) versus ≥6 (adequate prosocial behavior). Median (interquartile range, IQR) for continuous variables; *P* value was calculated by the Mann–Whitney U test. N (%) for categorical variables; *P* value was calculated by the chi-square test or Fisher's exact test as appropriate. $P < 0.05$ was considered statistically significant. Abbreviations: PB, prosocial behavior score; h/d, hours/day; CNY, Chinese Yuan (to convert to US dollar, multiply by 6.80).

Table S5 Sensitivity analysis using multiple imputation for missing or implausible age data (N = 22,609).

Sleep duration (h/d)	TDS >14		PB <6	
	**OR (95%CI)**	***P*-Value**	**OR (95%CI)**	***P*-Value**
10–13	Ref.		Ref.	
8–<10	1.48 (1.36, 1.60)	<0.0001	1.29 (1.22, 1.37)	<0.0001
<8	3.19 (2.69, 3.81)	<0.0001	2.42 (2.05, 2.87)	<0.0001

Adjusted for all covariates as in Table 2 Model III. TDS >14 indicates elevated risk for mental health problems; PB <6 indicates low prosocial behavior.
Abbreviations: OR, odds ratio; CI, confidence interval; h/d, hours/day.

Table S6 Sensitivity analysis excluding children with extreme short sleep duration (<8 h/d) for the association between sleep duration and mental health outcomes.

Sample	Sleep duration (h/d)	TDS >14		PB <6	
		**OR (95%CI)**	***P*-Value**	**OR (95%CI)**	***P*-Value**
Full sample (N = 21,366)	10–13 (n = 6140)	Ref.		Ref.	
	<10 (n = 15226)	1.49 (1.37, 1.62)	<0.0001	1.31 (1.23, 1.39)	<0.0001
After excluding <8 h/d (N = 20,797)	10–13 (n = 6140)	Ref.		Ref.	
	8–<10 (n = 14657)	1.44 (1.33, 1.57)	<0.0001	1.28 (1.21, 1.36)	<0.0001

Adjusted for all covariates as in Table 2 Model III. TDS >14 indicates elevated risk for mental health problems; PB <6 indicates low prosocial behavior. Abbreviations: OR, odds ratio; CI, confidence interval; h/d, hours/day.
